# Increased expression of the gene encoding stearoyl-CoA desaturase 1 in human bladder cancer

**DOI:** 10.1007/s11010-018-3306-z

**Published:** 2018-02-02

**Authors:** M. Presler, A. Wojtczyk-Miaskowska, B. Schlichtholz, A. Kaluzny, M. Matuszewski, A. Mika, T. Sledzinski, J. Swierczynski

**Affiliations:** 1 Department of Biochemistry, Medical University of Gdansk, Debinki 1, 80-211 Gdansk, Poland; 2 Department of Urology, Medical University of Gdansk, Smoluchowskiego 17, 80-214 Gdansk, Poland; 3 Department of Pharmaceutical Biochemistry, Medical University of Gdansk, Debinki 1, 80-211 Gdansk, Poland; 4 Department of Environmental Analysis, Faculty of Chemistry, University of Gdansk, Wita Stwosza 63, 80-308 Gdansk, Poland; 5 State School of Higher Vocational Education in Koszalin, Lesna 1, 75-582 Koszalin, Poland

**Keywords:** *SCD1*, *FASN*, *ELOVL6*, *SREBP1*, Bladder cancer

## Abstract

Bladder cancer is a common disease and a significant cause of death worldwide. There is thus great interest in identifying a diagnostic and prognostic biomarker, as well as gaining an understanding of the molecular basis of bladder cancer. Stearoyl-CoA desaturase 1 gene (*SCD1*) is highly overexpressed in many human cancers. However, the expression of *SCD1* has not yet been investigated in patients with bladder cancer. Here, we document that (a) the *SCD1* is highly overexpressed in human bladder cancer; (b) high expression of *SCD1* is more frequently observed in the late stage of disease and patients with lymph node metastasis; (c) bladder cancer patients with a higher SCD1 mRNA level have a poorer survival rate than those with normal *SCD1* expression. Overall, this is the first report to indicate an association between SCD1 mRNA level and clinical indicators of human bladder cancer. Our study has provided evidence supporting the potential role of *SCD1* as a biomarker for human bladder cancer prognosis.

## Introduction

Alteration in lipid metabolism is a biochemical hallmark of malignancy [1–5]. Over the past decade, numerous papers have implicated the gene expression and enzyme activity of stearoyl-CoA desaturase 1 (*SCD1*) in the pathogenesis of cancer [1, 6–8]. SCD1 catalyzes the conversion of saturated acyl-CoA (mainly 16:0, palmitoyl-CoA and 18:0, stearoyl-CoA) to monounsaturated acyl-CoA (16:1n-7, palmitoleoyl-CoA and 18:1n-9, oleoyl-CoA, respectively) leading to an alteration of the saturated-to-monounsaturated fatty acid ratio in the tissue [9]. It has been shown that the elevated expression of the gene encoding SCD1 associated with increased levels of monounsaturated fatty acid in blood and tumor tissues is a metabolic feature of many cancer cells [1].

SCD1 activity is regulated mainly by the rate of the transcription of a gene encoding the enzyme [10]. In liver, adipose tissues, and cancer cells, the transcription of the *SCD1*, as with most of the genes encoding lipogenic enzymes, is regulated mainly by the sterol regulatory element binding protein 1c (SREBP1c), sometimes called sterol regulatory binding transcription factor 1 (SREBF1) [11–14]. SREBP1 is involved in the regulation of cancer cell proliferation, apoptosis, migration, and invasion [15, 16]. Recently, SREBP1 has been proposed as a prognostic marker of breast cancer [16]. Overexpression of the *SREBP1* has been also observed in endometrial, prostate, gastric, and pancreatic cancers [17–20]. Finally, SREBP1 and SCD1 are potential targets for cancer therapy [5, 21].

Alterations in fatty acid oxidation and biosynthesis have been also observed in bladder cancer [2]. In our previous study, we showed that the activities of fatty acid synthase (FASN), ATP citrate lyase (ACLY), and the dehydrogenases of pentose phosphate pathway are significantly higher in human bladder cancer than in adjacent benign tissue. Coordinated upregulation of FASN, ACLY, and pentose phosphate dehydrogenases that provide NADPH for palmitate biosynthesis, suggest that the rate of lipogenesis is also elevated in human bladder cancer. Moreover, we found that the activity of glycerol 3-phosphate dehydrogenase, the enzyme which creates a link between glycolysis (carbohydrate metabolism) and lipid biosynthesis by providing 3-phosphoglycerol, is higher in human bladder cancer tissue than in adjacent benign tissue [22]. Furthermore, emerging data suggest that overexpression of the *FASN* is associated with tumor aggressiveness and migratory capacity of bladder cancer cells, as well as with resistance to chemotherapy.

Relatively little information is available regarding *SCD1, SREBP1*, and *ELOVL6* (encoding elongase 6) expression in human bladder cancer. To our best knowledge, the *SCD1* expression has so far been evaluated only in human bladder cell lines [13]. The associations between SCD1 mRNA level and clinical and pathological features, as well as the prognostic significance of *SCD1* expression, are unknown in human bladder cancer. Additionally, the association between the SCD1 mRNA level and the expression of *SREBP1* and *ELOVL6* remains to be elucidated in human bladder cancer.

Our study thus aimed to investigate SCD1 mRNA levels in human bladder cancer and the relationship between *SCD1* expression and clinical and pathological features, including stage and lymphatic metastasis.

## Materials and methods

### Patients

We examined 39 patients (median age 62 years) with transitional cell carcinoma (TCC) who underwent transurethral resection of bladder tumor (TURBT) or radical cystectomy at the Department of Urology, Medical University of Gdansk (Table 1). The Local Ethics Committee of the Medical University of Gdansk approved the use of tissue samples from patients in the study. A total of 39 bladder cancer and adjacent noncancerous tissues were frozen in liquid nitrogen immediately after surgery and stored at − 80 °C prior to use.

**Table 1:** Demographic, clinical, and pathological characteristics of patients

Variable category	Case (N) (%)
Sex	
Female	10 (26)
Male	29 (74)
Age	
≤ 62	23 (59)
> 62	16 (41)
TURBT	15 (38)
Radical cystectomy	24 (62)
Grade (1973 WHO)	
G1	11 (28)
G2	12 (31)
G3	13 (33)
Not known	3 (8)
TNM stage	
Ta	6 (15)
T1	9 (23)
T2	5 (13)
T3	12 (31)
T4	7 (18)
Lymph node status	
Nx	16 (41)
N0	13 (33)
N1	7 (18)
N2	2 (5)
N3	1 (3)

### qPCR analysis

To analyze *SCD1, FAS, ELOVL6*, and *SREBP1* expression in bladder tissues, the total RNA was isolated using GenElute Mammalian Total RNA Miniprep Kit (Sigma Aldrich). RNA quality was verified with Experion RNA Analysis Kit (Bio-Rad). For cDNA synthesis, 1 µg of total RNA was reverse transcribed using Thermo Scientific First Strand cDNA Synthesis Kit. DNase (Thermo Scientific) treatment was used to remove residual DNA contamination. Amplification reactions were performed on a CFX96 Touch Real-Time PCR Detection System (Bio-Rad) using Hot FirePol EvaGreen qPCR Mix Plus (Solis Biodyne). Gene-specific primers were designed as follows: *SCD1* F-5′-TCTTCTCTCACGTGGGTTGG-3′; *SCD1* R-5′-AGCCAGGTTTGTAGTACCTCCT-3′; *FASN* F-5′-CGGAGGCATCAACCCAGATT-3′; *FASN* R-5′-CTGTAGCCCACGAGTGTCTC-3′; *ELOVL6* F-5′-ATGGATGCAGGAAAACTGGAAG-3′; *ELOVL6* R-5′-ATTCATTAGGTGCCGACCAC-3′; *SREBP1* F-5′-GGACAGCAAGGCAAAGCCA-3′; *SREBP1* R-5′-GGTAGACGCTGGTGGTATCTG-3′; *PPIA* (encoding cyclophilin A) F-5′-ATCTGCACTGCCAAGACTGAG-3′; *PPIA* R-5′-GAAGGAATGATCTGGTGGTTAAGA-3′. The Livak and Schmittgen method, with calculations providing 2^−ΔΔCt^ values, was used to evaluate the relative gene expression level [23]. For this purpose, the expression of a housekeeping gene, *PPIA* was also determined in order to normalize the results. Additionally, noncancerous tissue was used as a calibrator [24]. By direct comparison of the cancerous and noncancerous specimens derived from the same individual, we obtained a ‘subtractive’ effect whereby changes in the expression of selected genes were distinguishable. Each analysis was performed in duplicate. The relative expression of mRNA at a level below two was considered to be unchanged.

### Statistics

Statistica 12 was used for statistical analysis. The differences between the Ta/T1 and T2–T4 groups were determined by applying the Mann–Whitney *U* test. In addition, the Kruskal–Wallis test was used to compare differences between the Ta, T1, T2, T3, and T4 groups and the Mann–Whitney *U* test was performed to check changes between the medians for each pair of tumor stages. The association between the mRNA relative expressions of two genes was analyzed using Pearson’s correlation coefficient. The statistically significant difference for the Kaplan–Meier survival probability was verified by the log-rank test. *P* values ≤ 0.05 were considered statistically significant.

## Results

### Association of SCD1 mRNA levels with tumor stage

**Fig. 1:**
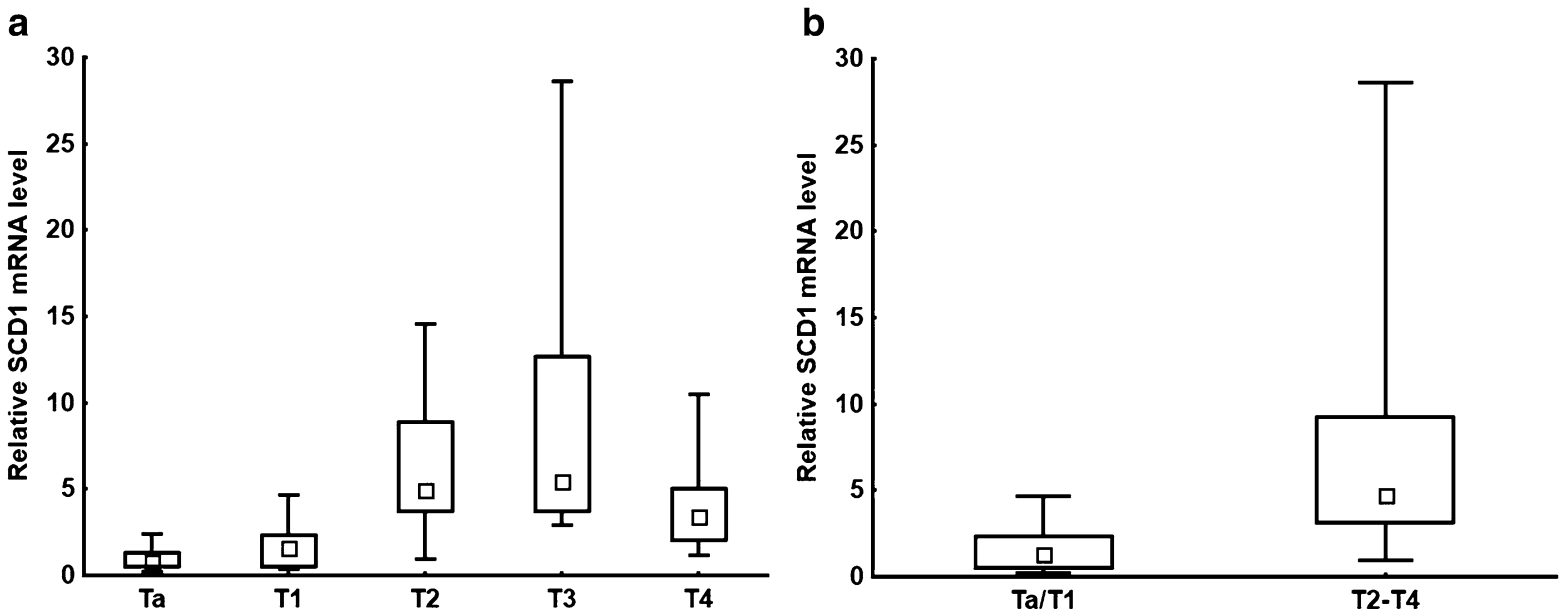
**a** The relative SCD1 mRNA level for Ta, T1, T2, T3, and T4 groups; Ta–T2 *P* = 0.036; Ta–T3 *P* < 0.001; Ta–T4 *P* = 0.012; T1–T2 *P* = 0.045; T1–T3 *P* < 0.001; T1–T4 *P* = 0.044. **b** Relative SCD1 mRNA level for Ta–T1 and T2–T4 groups; *P* < 0.001. The median is shown in the box and the upper and the lower boundaries indicate 50% of results within the box. The whiskers extend to minimum and maximum values

Figure 1a shows a progressive increase in SCD1 mRNA levels, ranging from Ta to T3 stage. Low SCD1 mRNA levels were characteristic of the Ta and T1 stages of bladder cancer patients (Fig. 1a). Surprisingly, though the Mann–Whitney *U *test did not reveal any statistically different median values between SCD1 mRNA levels in T3 and T4 patients (*P* = 0.083), the somewhat noticeable downward trend may be observed in the most advanced stage of bladder cancer. However, the relatively small number of patients classified as stages T3 and T4 suggested that these results need clarification through further study. Considering the level of SCD1 mRNA and the relatively small number of patients in the T1, T2, T3, and T4 groups, respectively, all the examined bladder cancer patients were classified into (a) the Ta/T1 group (that represented a low SCD1 mRNA level), or (b) the T2–T4 group (that showed significantly higher SCD1 mRNA level) (Fig. 1b). It should be emphasized that *SCD1* expression median values were significantly different for the Ta/T1 group and the T2–T4 group (*P* < 0.001) (Fig. 1b). Higher relative SCD1 mRNA levels were observed in patients with more advanced stage. Although there was a trend showing an increasing mRNA level for *SCD1, ELOVL6, FASN, SREBP1* genes towards higher grade, no statistically significant changes were observed.

### Association of ELOVL6, FASN, and SREBP1 mRNA levels with tumor stage

**Fig. 2:**
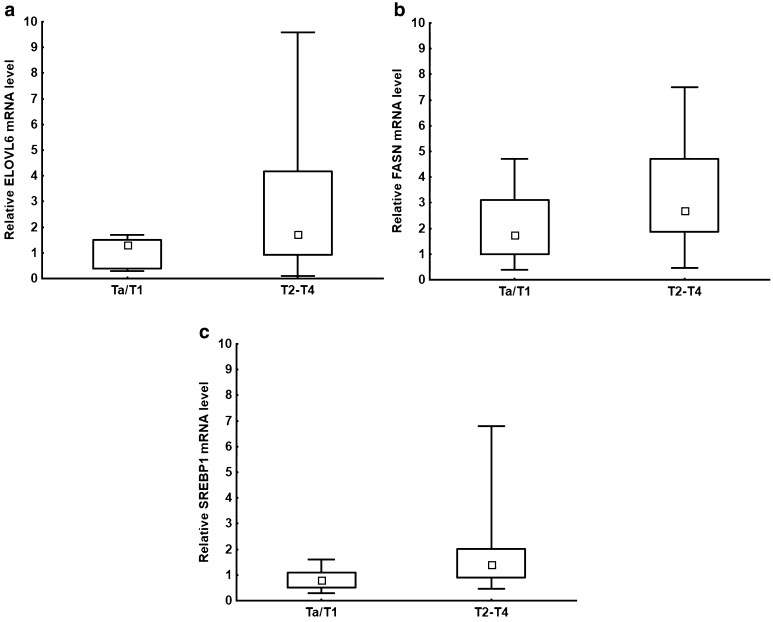
**a** Relative ELOVL6 mRNA level for Ta–T1 and T2–T4 groups; *P* = 0.036. **b** Relative FASN mRNA level for Ta–T1 and T2–T4 groups; *P* = 0.04. **c** Relative SREBP1 mRNA level for Ta–T1 and T2–T4 groups; *P* = 0.005. The median is shown in the box and the upper and the lower boundaries indicate 50% of results within the box. The whiskers extend to minimum and maximum values

As with SCD1, a progressive increase in the level of ELOVL6 mRNA was found in bladder cancer. In the T2–T4 stages of bladder cancer, a significant increase in ELOVL6 mRNA was found (*P* = 0.036) (Fig. 2a). Furthermore, a positive correlation was found between ELOVL6 and SCD1 mRNA levels (*r* = 0.32; *P* = 0.048).

*FASN* expression in bladder cancer patients also revealed increased FASN mRNA levels in the T1, T2, T3, and T4 groups, but not in Ta. In particular, more than half of T1–T4 patients showed elevated FASN mRNA levels over 2 (Fig. 2b). It is noteworthy that many patients with increased *FASN* expression also had elevated *SCD1* expression with highly significant positive correlation (*r* = 0.65; *P* < 0.001).

A difference was seen in SREBP1 mRNA expression (*P* = 0.005) between the Ta/T1 and T2–T4 groups, with results representing expressions above 2 in stages T2–T4 (Fig. 2c). Additionally, those patients who had even mildly increased SREBP1 mRNA levels usually showed heightened *SCD1* gene expression. Consequently, a statistically significant correlation was found between SCD1 and SREBP1 mRNA levels (*r* = 0.7; *P* < 0.001). A similar relation was also seen for FASN and SREBP1 mRNA levels (*r* = 0.46; *P* = 0.005).

### Association of SCD1 and FASN mRNA expression with lymph node metastasis and survival probability of bladder cancer patients

**Fig. 3:**
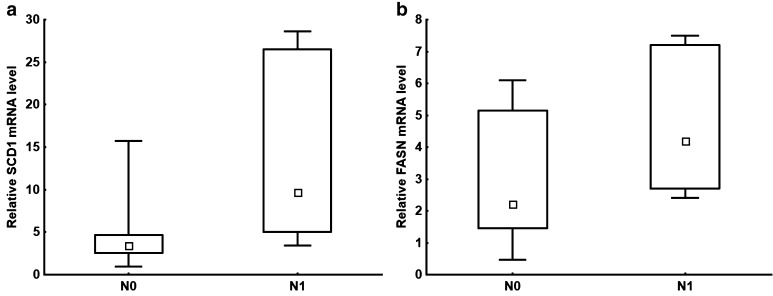
Relative levels of **a** SCD1 mRNA corresponding to no lymph node metastasis (N0) and the presence of metastasis in a single lymph node (N1); *P* = 0.022; **b** FASN mRNA corresponding to no lymph node metastasis (N0) and the presence of metastasis in a single lymph node (N1); *P* = 0.069. The median is shown in the box and the upper and the lower boundaries indicate 50% of results within the box. The whiskers extend to minimum and maximum values

A statistically significant difference (*P* = 0.022) was observed in the SCD1 mRNA level in the bladder cancer tissue of patients with the absence and presence of metastasis in one of the lymph nodes (Fig. 3a). The mean was around 2.5 times greater in the cancer present in a single lymph node than in the absence of cancer in any lymph node. A slight difference was also seen for *FASN* gene expression (*P* = 0.069) (Fig. 3b).

**Fig. 4:**
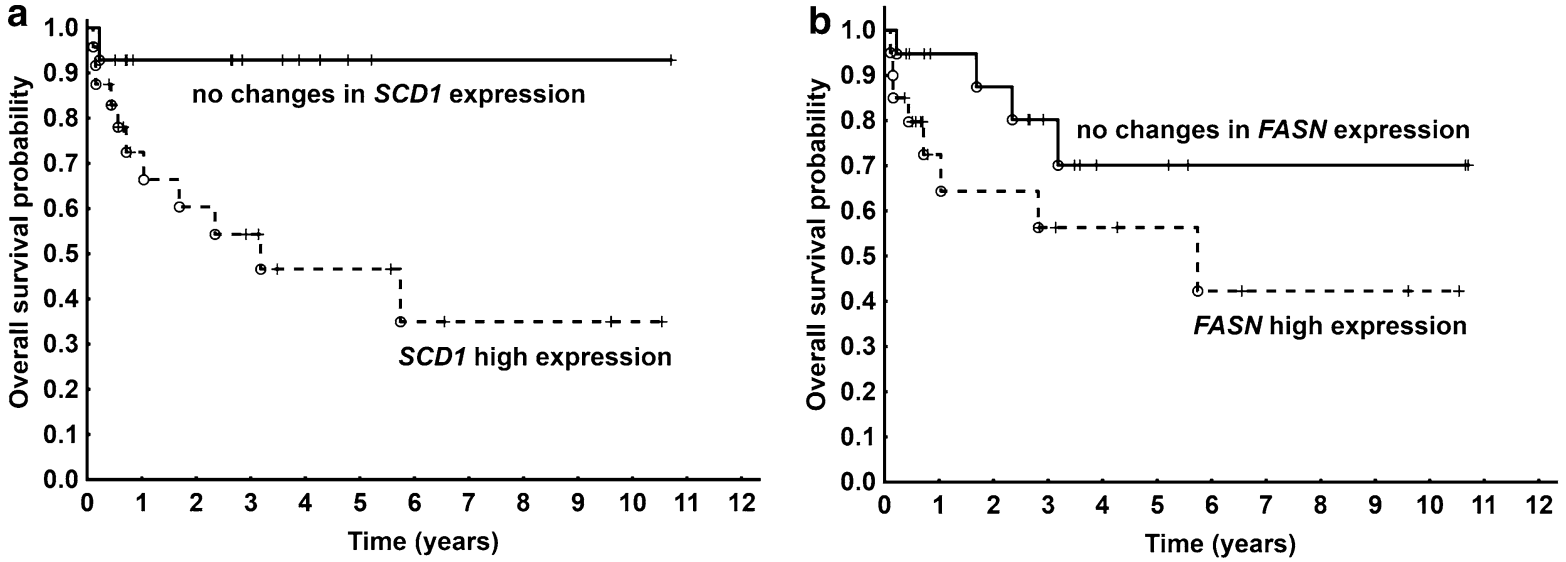
Kaplan–Meier survival curves for patients with **(a)** SCD1 mRNA high expression or with no changes; *P* = 0.021; **(b)** FASN mRNA high expression or with no changes; *P* = 0.161

Interestingly, Kaplan–Meier analysis for *SCD1* expression showed more than a 90% survival probability for bladder cancer patients who did not have any (or had small) changes in the expression of this gene (Fig. 4a). On the other hand, patients with high SCD1 mRNA expression level demonstrated significantly lower overall survival probabilities of up to almost 30% within 6 years (*P* = 0.021).

Although there were no statistically significant changes (*P* = 0.161) in the overall survival probability between patients with no changes in *FASN* gene and with increased *FASN* expression, it can be concluded that higher expression (mostly in T2–T4 patients) was related to shorter survival (Fig. 4b).

**Fig. 5:**
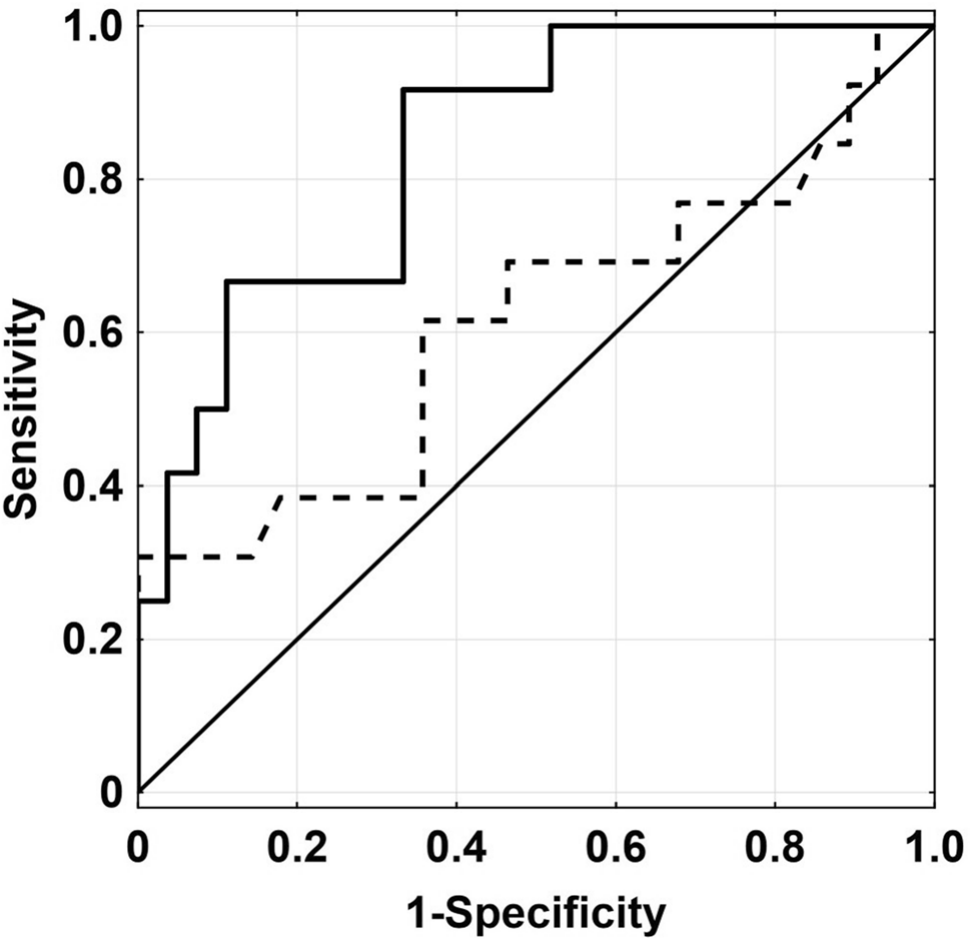
ROC curves for **a** SCD1 mRNA level (continuous line); AUC = 0.843; CI 0.72–0.97; *P* < 0.001; **b** FASN mRNA level (dotted line); AUC = 0.613; CI 0.41–0.82; *P* < 0.278

Receiver operating characteristic (ROC) curves were prepared for patients who completed survival in relation to *SCD1* expression and *FASN* expression. As a result, *SCD1* expression appeared to have a better AUC of 0.843 than did *FASN* (AUC = 0.613), as well as statistical significance (*P* < 0.001) (Fig. 5).

## Discussion

In this study, we found for the first time that the gene encoding SCD1 is highly overexpressed in human bladder cancer, as compared to adjacent benign tissue. Moreover, this study showed a progressive increase in SCD1 mRNA levels from the Ta to the T3 stage. Additionally, we found a significantly higher level of SCD1 mRNA in bladder cancer patients with lymph node metastasis. Kaplan–Meier analysis showed that human bladder cancer patients with a higher level of SCD1 mRNA had poorer survival rates than those without elevated *SCD1* expression. In general, our findings concerning human bladder cancer are consistent with previous data reporting that the level of *SCD1* expression is associated with tumorigenesis and prognosis of numerous cancers, including breast, prostate, colon, lung, kidney, thyroid, and lymphoma [1]. Our results suggest that SCD1 mRNA level may serve as a novel potential biomarker for human bladder cancer prognosis.

Moreover, we confirmed in this study previously reported results showing the elevated activity of FASN and the upregulation of gene encoding FASN in human bladder cancer [22, 25–28]. Interestingly, the SCD1 mRNA levels at each stage of the disease were significantly higher than the FASN mRNA level in human bladder cancer. Thus, it can be supposed that the level of SCD1 mRNA could be a more useful marker of human bladder cancer prognosis than the level of FASN mRNA, which is commonly studied in many cancer cells, including bladder cancer. This is confirmed by the Kaplan–Meier and ROC analyses.

Finally, we have shown the upregulation of *SREBP1* and *ELOVL6* in human bladder cancer. The correlation between SREBP1 and SCD1 (and other lipogenic enzymes) mRNA levels suggests that SREBP1 plays an important role in the regulation of lipogenesis, especially in *SCD1* transcription in human bladder cancer. To our best knowledge, this is the first study to investigate SREBP1 and ELOVL6 mRNA levels in bladder cancer and their association with the clinical and pathological features of patients with bladder cancer. In this respect, our findings are consistent with previous data that *SREBP1* is highly expressed in many cancers, including pancreatic cancer, breast cancer, endometrial cancer, prostate, and gastric cancer, and is associated with tumorigenesis [16–20]. However, it should be emphasized that the level of SREBP1 mRNA is several-fold lower than that of SCD1 or FASN mRNA in human bladder cancer. Thus, the clinical significance of the SREBP1 mRNA assay as a potential marker of human bladder cancer prognosis is rather negligible.

Although there is a progressive increase in the level of SCD1 mRNA between stages Ta and T3, there was also a decrease in SCD1 mRNA levels in T4 compared to T3, though this did not reach statistical significance. However, it should be noted that SCD1 mRNA levels in the T4 stage were still significantly higher than in the Ta and T1 stages (Fig. 1a). A similar result was observed in FASN and SREBP1 mRNA levels (not shown). Based on the data presented here, it can be supposed that a reduction in *SCD1* (and other lipogenic genes) transcription may occur in the patients in group T4. Interestingly, the decrease in levels of protein biomarkers, including adipocyte-type fatty acid-binding protein (aFABP), glutathione S-transferase µ (GST µ), and 15-hydroxyprostaglandin dehydrogenase (PGDH), as well as cytokeratin 13 (CK13), has been described in high-grade human bladder cancer [29, 30]. Overall, these results suggest that not only the *SCD1, FASN*, and *SREBP1*, but probably also other genes, are downregulated in high-grade human bladder cancer. Similar changes, namely the upregulation (up to stage T3) and then downregulation of *aFABP* and *PGDH* gene expression and lipogenic enzyme in human bladder cancer, are also worthy of interest [30].

The molecular mechanism of SCD1 in the proliferation promotion of bladder cancer cells is not clear. The proliferation rate of cancer cells is much faster than that of adjacent benign cells. Subsequently, greater quantities of lipids are required for cancer cell proliferation. It is likely that the coordinated upregulation of *FASN* and *SCD1*, regulated by SREBP1, is contributing to the biosynthesis of fatty acids necessary for bladder cancer cell proliferation. The correlation between (a) SCD1 and SREBP1 mRNA levels; (b) FASN and SREBP1 mRNA levels; and (c) SCD1 and FASN mRNA levels at least partly support this assumption. This is the simplest explanation for the upregulation of all genes encoding lipogenic enzymes. However, the reason for the upregulation of the SREBP1—a master regulator of lipogenesis—requires further investigation. In some cancer cells, SREBP1 is regulated not only by sterols, but also by the phosphatidylinositol-4,5-bisphosphate 3-kinase/Akt oncogenic signaling pathway [31, 32]. It is possible that a similar mechanism occurs in human bladder cancer cells.

Other molecular mechanisms could also explain the role of SCD1 in the proliferation of bladder cancer cells. According to Igal and co-workers, SCD1 may control cancer cell proliferation via modulation of the epidermal growth factor (EGFR → Akt/ER) signaling pathway [33]. Du et al., studying an association between fibroblast growth factor receptor 3 (FGFR3) and SCD1 in human bladder cancer cell lines, concluded that FGFR3 stimulates SCD1 activity to promote tumor growth [13].

A few limitations to our study should be addressed. Firstly, our study involved only 39 patients. This limitation results from the study design, which meant that only samples stored in a single center were analyzed retrospectively. A study in a greater population is thus necessary to clearly assess the association between SCD1 mRNA level and bladder cancer progression and poor survival. However, it seems that our results are reliable and consistent with the data reported previously, indicating that *SCD1* (along with other genes encoding lipogenic enzymes) is highly overexpressed in numerous cancers, and that the level of the expression is associated with clinical features. Secondly, we measured only mRNA levels, so protein levels and SCD1 activity need to be investigated in the future. Nevertheless, as mentioned in the Introduction, SCD1 activity is regulated mainly by the rate of transcription of the gene encoding the enzyme.

In conclusion, we report that the highest expression of *SCD1* in human bladder cancer is associated with (a) stage; (b) lymph node metastasis; and (c) worse survival. Moreover, this study shows the upregulation of the expression of the gene encoding SREBP1, which is the transcription factor regulating the transcription of genes encoding lipogenic enzymes, including SCD1. Overall, it is the first report that indicates the association between the SCD1 mRNA level and the clinical factors of human bladder cancer. Our study suggests a potential role of SCD1 as a biomarker for the prognosis of human bladder cancer.
